# Network Pharmacology-Based Systematic Analysis of Molecular Mechanisms of Dingji Fumai Decoction for Ventricular Arrhythmia

**DOI:** 10.1155/2021/5535480

**Published:** 2021-05-08

**Authors:** Yi Liang, Bo Liang, Xin-Rui Wu, Wen Chen, Li-Zhi Zhao

**Affiliations:** ^1^Southwest Medical University, Luzhou, China; ^2^Nanjing University of Chinese Medicine, Nanjing, China; ^3^Hospital (T.C.M.) Affiliated to Southwest Medical University, Luzhou, China

## Abstract

**Background:**

Dingji Fumai Decoction (DFD), a traditional herbal mixture, has been widely used to ventricular arrhythmia (VA) in clinical practice in China. However, research on the bioactive components and underlying mechanisms of DFD in VA is still scarce.

**Methods:**

Components of DFD were collected from TCMSP, ETCM, and literature. The chemical structures of each component were obtained from PubChem. Next, SwissADME and SwissTargetPrediction were applied for compounds screening and targets prediction of DFD; meanwhile, targets of VA were collected from DrugBank and Online Mendelian Inheritance in Man (OMIM). Then, the H-C-T-D network and the protein-protein interaction (PPI) network were constructed based on the data obtained above. CytoNCA was utilized to filter hub genes and VarElect was used to analyze the relationship between genes and diseases. At last, Metascape was employed for systematic analysis on the potential targets of herbals against VA, and AutoDock was applied for molecular docking to verify the results.

**Results:**

A total of 434 components were collected, 168 of which were qualified, and there were 28 shared targets between DFD and VA. Three function modules of DFD were found from the PPI network. Further systematic analysis of shared genes and function modules explained the potential mechanism of DFD in the treatment of VA; molecular docking has verified the interactions.

**Conclusions:**

DFD could be employed for VA through mechanisms, including complex interactions between related components and targets, as predicted by network pharmacology and molecular docking. This work confirmed that DFD could apply to the treatment of VA and promoted the explanation of DFD for VA in the molecular mechanisms.

## 1. Introduction

In recent years, cardiovascular diseases are the leading cause of death in China [1]. All cardiac conditions, especially ischemic heart disease, can lead to arrhythmias [2]. Among all arrhythmias, ventricular arrhythmia (VA) has the highest mortality. VA is a common but life-threatening disease, mainly including ventricular premature contraction, ventricular tachycardia, ventricular flutter, and ventricular fibrillation, with a clinical presentation that ranges from no symptoms to cardiac arrest [2]. VA is usually generated by all-caused enhanced automaticity or abnormal automaticity, myocardial ischemia, delayed afterdepolarizations, and structural heart disease with reentry [3–5]. To prevent adverse events of VA, millions of patients were treated with beta-receptor-blockers, INa antagonists, IKr antagonists, nondihydropyridine calcium antagonists, and other drugs suggested by the 2017 American Heart Association/American College of Cardiology/Heart Rhythm Society guideline [2], but the control of VA is still far from ideal.

Traditional Chinese medicine (TCM) has a clinical-based development history of over 2000 years [6, 7]. Some researchers mentioned that TCM could be used to treat a variety of diseases, including VA [8–10]. Besides, related studies suggested that, compared with Western medicine only, patients suffering from various diseases can benefit more from a TCM and Western medicine combined therapy strategy [11–22]. Moreover, several TCM, such as Shensong Yangxin Capsule [9] and Wenxin Keli [23], have been transformed into commercial products for the treatment of cardiovascular-related diseases. Such convenient approaches greatly promoted the development of TCM worldwide.

**Figure 1:**
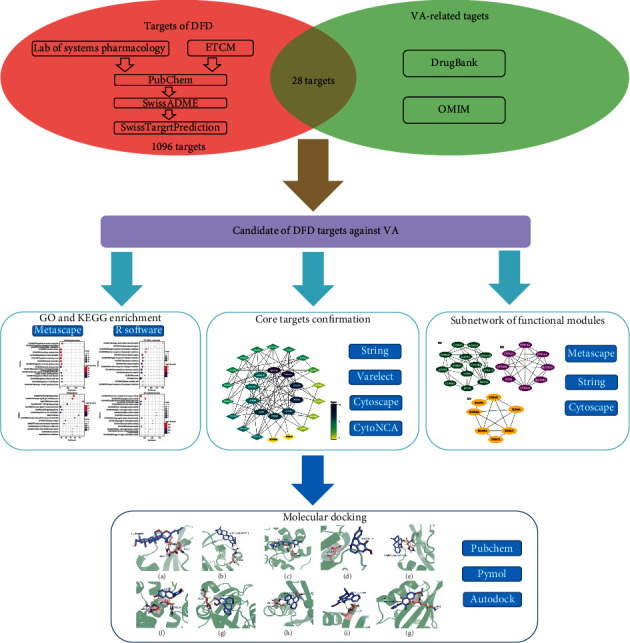
Flowchart of this work.

Dingji Fumai Decoction (DFD), consisting of *Chuanxiong Rhizoma* (Chuanxiong), *Jujubae Fructus* (Dazao), *Poria cocos* (Schw.) Wolf (Fuling), *Cinnamomi Ramulus* (Guizhi), *Silktree Albizia Bark* (Hehuanpi), *Osdraconis* (*Fossiliaossiamastodi*) (Longgu), *Ostrea Gigas Thunberg* (Muli), *Ziziphi Spinosae Semen* (Suanzaoren), *Radix Polygalae* (Yuanzhi), and *Licorice* (Gancao), is widely used for VA and obtains encouraging results [24], and previous experiments revealed that one of the underlying mechanisms is class I antiarrhythmic property [25]. To make DFD more recognized, it is essential to ensure the efficacy and safety of DFD. Since multiple components are contained in DFD, it can generate interactions on multiple targets. Based on the theory of network pharmacology, we constructed network relationships between “component-target pathways” to explore the mechanisms of drugs or herbs [26]. Here, we analyzed the mechanisms of DFD in the treatment of VA systematically (Figure 1). At first, we obtained its components and potential targets against VA. Then, the protein-protein interaction (PPI) of potential targets against VA was constructed. Next, a systematic analysis of potential targets and biofunctional modules was conducted. Meanwhile, the interactions between the components of DFD and key targets were confirmed using molecular docking.

## 2. Methods

### 2.1. Chemical Structures Construction

The Traditional Chinese Medicine Systems Pharmacology database and analysis platform (TCMSP) [27] and the Encyclopedia of Traditional Chinese Medicine (ETCM) [28] are web-based herb databases, providing comprehensive and standardized information for the commonly used herbs. In this study, the components of each herb in DFD were obtained from TCMSP, ETCM, and published literature. To make the components recognizable for the subsequent analysis work, after removing duplicates, the structure of each component was collected from PubChem [29].

### 2.2. Gastrointestinal Absorption (GA) and Drug-Likeness (DL) Prediction

Increasingly researchers found that TCM despite their impressive *in vitro* findings demonstrate less or negligible *in vivo* activity, resulting in poor absorption and hence poor bioavailability [30]. The absorption, distribution, metabolism, and excretion (ADME) of the drug must be considered by the researcher and developer [31]. Bioabsorption is highly multifactorial but is primarily driven by GA [32]. Besides, DL assesses qualitatively the chance for a molecule to be an oral drug with respect to bioavailability [31]. It was constructed that the estimation of ADME before the drug development studies reduces the possibility of failure [33]. In the mechanism explaining DFD, GA and DL were evaluated using SwissADME, a free tool that could evaluate DL, GA, pharmacokinetics, and medicinal chemistry friendliness of small molecules [31]. After uploading the structure of each compound to SwissADME, if the prediction results of the component suggested high possibility of both GA and DL, it met our inclusion criteria and was adopted for the next screening [31].

### 2.3. Target Prediction and Verification

In the treatment of diseases, not all absorbable components work; therefore, we filtered out the components with bioactive components from all absorbable components using SwissTargetPrediction, an online tool that can evaluate compounds with a score by fitting a multiple logistic regression on various subsets of known actives to weight structure similarity parameters [34]. Here, we uploaded the structure of each component to SwissTargetPrediction to predict potential targets of DFD, and all possible targets were adopted.

Online Mendelian Inheritance in Man (OMIM) is a knowledge base providing the latest information of human genes [35], and DrugBank is a freely available and comprehensive web resource providing drug-target and drug interaction information [36]. Taking “ventricular arrhythmia” or “arrhythmia of ventricular origin” as a keyword, we obtained VA-related targets from OMIM and DrugBank. Taking the intersection of DFD and VA targets, the common targets between DFD and VA were considered the therapeutic targets of DFD against VA, as described previously [37].

PPI is one of the cores of cellular processing. The analysis of PPI makes the interactions of proteins clear and helps to explain the function of possible protein complexes or functional modules [38]. STRING is a web database providing online analysis of PPI [38]. After uploading the common targets to STRING, we constructed the PPI network. Then, the result was imported to Cytoscape (version 3.8.0) for further analysis [39]. CytoNCA plugin in Cytoscape was applied to analyze centrality of certain targets and evaluate protein interaction networks [40].

The VarElect online tool can analyze direct and indirect links between genes and diseases [41]. In this study, the link relationships of potential targets of DFD against VA were analyzed with VarElect; the results helped determine which targets will be included in the next molecular docking.

### 2.4. Biology Functional Analysis

Since Gene Ontology (GO) and Kyoto Encyclopedia of Genes and Genomes (KEGG) can contribute to the interpretation of system-level data and enable new discoveries [42], in this study, Metascape was employed for GO and KEGG analysis to further explore the complex mechanism of DFD in the treatment of VA. Metascape is a web-based platform providing gene annotation, functional enrichment, and interactome analysis services; monthly database update could keep our analysis results up to date [42]. In our work, GO and KEGG terms with *P* < 0.01 were considered significantly enrichment analyses.

### 2.5. Molecular Docking

Molecular docking was used to assess interactions between components and hub targets; the 4 hub targets connected to VA closely were included. The structures of these targets were collected from Protein Data Bank [43]. AutoDock and PyMOL were employed for molecular docking, PyMOL was used to remove the water molecules and isolate proteins of the moleculars [44]. AutoDock was used to add the hydrogen and calculate Gasteiger charges of the moleculars and add the hydrogen for the ligands [45]. At last, molecular docking was conducted using AutoDock to assess the binding energy; the lower the binding energy, the more stable the docking modules [45].

## 3. Results

### 3.1. Chemical Structures Construction

After searching TCMSP, ETCM, and literature [27, 28], a total of 434 compounds were collected, including 92, 73, 76, 38, 19, 70, 12, 36, and 18 compounds in *Licorice, Chuanxiong Rhizoma, Jujubae Fructus, Poria cocos* (Schw.) Wolf*, Cinnamomi Ramulus, in Silktree Albizia Bark, Ostrea Gigas Thunberg, Ziziphi Spinosae Semen,* and *Radix Polygalae.* Later, the structure of each component was collected from PubChem.

### 3.2. GA and DL Prediction

The structures were uploaded to SwissADME; after screening the GA and DL and removing duplicates, 168 components qualified, including 80, 20, 27, 11, 4, 19, 4, 10, and 2 in *Licorice, Chuanxiong Rhizoma, Jujubae Fructus, Poria cocos* (Schw.) Wolf*, Cinnamomi Ramulus, Silktree Albizia Bark, Ostrea Gigas Thunberg, Ziziphi Spinosae Semen, and Radix Polygalae,* respectively. Interestingly, several qualified components are owned by more than one herbal; more information about the prediction results was placed in the Supplementary Materials. All qualified components were adopted for the next screening.

### 3.3. Target Prediction and Verification

**Figure 2:**
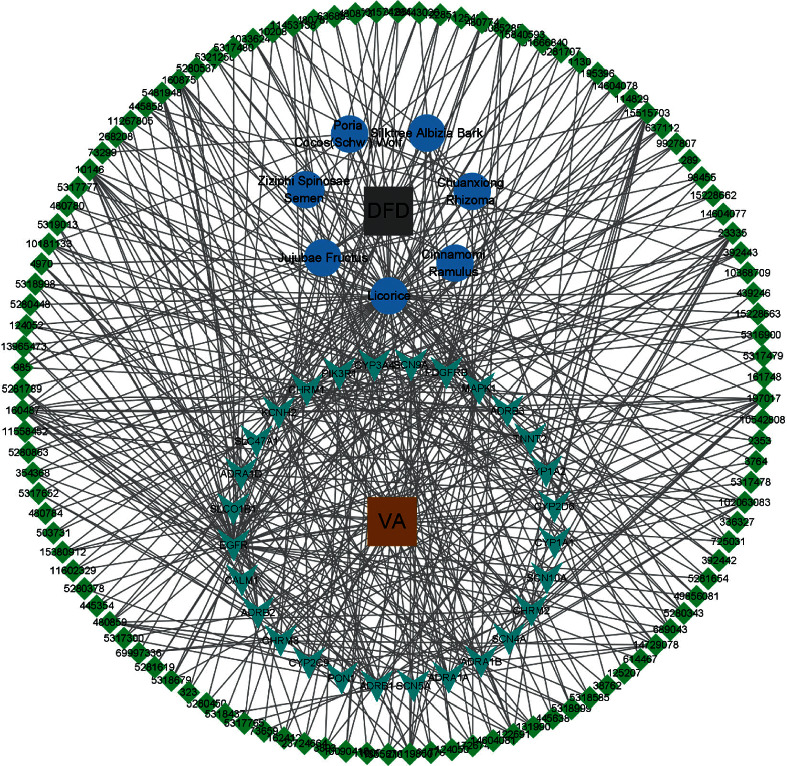
The H-C-T-D network of DFD. Grey and chocolate rectangles indicate DFD and VA. Blue round indicates the 7 herbal medicines comprising DFD. Green quadrilateral indicates the 110 active compounds and cyan shape V indicates the 28 shard targets, respectively.

After removal of duplicates, a total of 1096 potential targets of DFD were collected from SwissTargetPrediction. Meanwhile, a total of 260 known therapeutic targets for VA were obtained from OMIM and DrugBank. Taking the intersection of DFD and VA targets, there were 28 shared targets; based on the data obtained above, the Herb-Compound-Targets-Disease (H-C-T-D) network was constructed. The H-C-T-D network was composed of 147 nodes (DFD, VA, 7 herbals, 110 bioactive compounds, and 28 common targets) and 465 edges (Figure 2).

**Figure 3:**
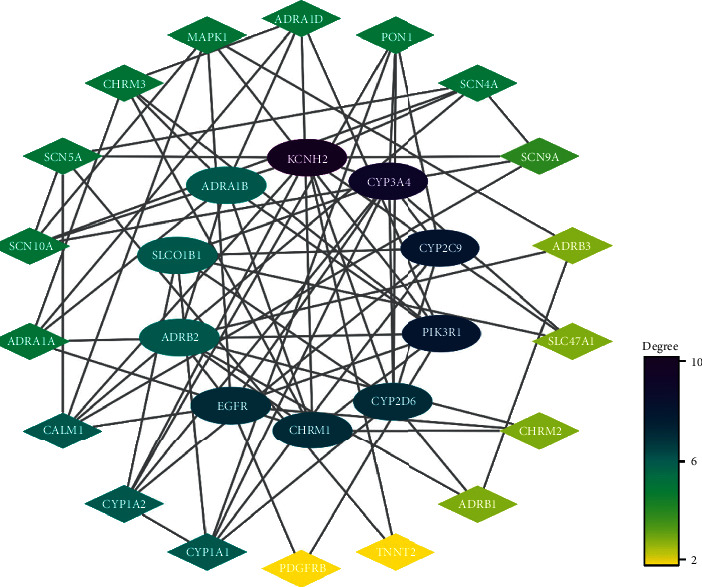
PPI network of potential targets of DFD against VA. Oval nodes on the inner circle indicate hub targets.

**Figure 4:**
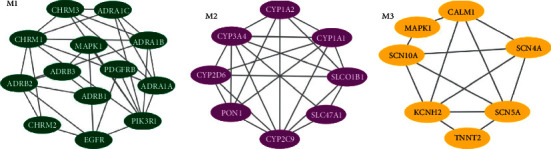
Three possible biofunctional modules divided from the PPI network.

In further analysis, all 28 common targets were uploaded to STRING to construct the PPI network; the results were imported to Cytoscape to calculate the degree value of each gene using CytoNCA plugin and reconstruct the PPI network according to the degree value (Figure 3). There were three possible biofunctional modules divided from the PPI network (Figure 4). After screening by CytoNCA, the top 10 targets were defined as hub targets; the designations and topological parameters of hub targets are shown in Table 1.

We analyzed the 28 common targets using VarElect to investigate the correlation between targets and VA, and the results suggest that 23 targets were related to VA directly, whereas 5 targets were related to VA indirectly (Table 2); among these targets, Potassium Voltage-Gated Channel Subfamily H Member 2 (KCNH2), Sodium Voltage-Gated Channel Alpha Subunit 5 (SCN5A), Troponin T2-Cardiac Type (TNNT2), and Calmodulin 1 (CALM1) have the highest score of correlation.

### 3.4. Biology Functional Analysis

**Figure 5:**
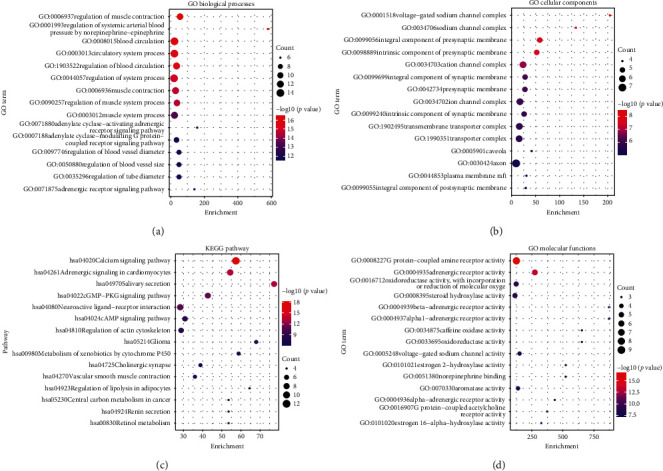
GO and KEGG enrichment analysis of potential targets.

The enrichment analysis of GO and KEGG of the 28 common targets was analyzed using Metascape, the results were ranked by −log 10 *(P value)*, and the top 14 of each enrichment item are shown in Figure 5; besides, the functional analysis of the three potential biofunction modules divided from PPI network is shown in Table 3.

In GO and KEGG enrichment analysis, terms with high enrichment scores suggest that the regulation of muscle contraction, regulation of systemic arterial blood pressure by norepinephrine-epinephrine, blood circulation, circulatory system process, adrenergic receptor activity, calcium signaling pathway, adenylate signaling in cardiomyocytes, cGMP-PKG signaling pathway, and neuroactive ligand-receptor interaction could be the most possible mechanisms of DFD in treating VA. For the 3 protein modules, Module 1 can regulate calcium signaling pathway, heart rate, and cAMP signaling pathway. Module 2 can regulate membrane depolarization during an action potential, striated muscle contraction, regulate adrenergic signaling in cardiomyocytes, and cation homeostasis. And Module 3 is more likely to possess the function of regulation of the metabolic process.

### 3.5. Molecular Docking

**Figure 6:**
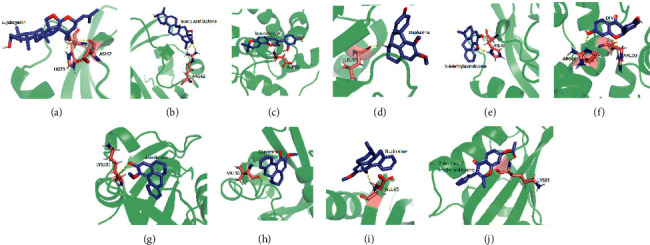
The top 10 binding energy docking modules.

Molecular docking was conducted to calculate binding energy between components and hub targets, KCNH2 (PDB ID:1BYW), SCN5A (PDB ID: 4DCK), TNNT2 (PDB ID: 1J1D), and CALM1 (PDB ID: 1CDL); the 4 genes connected closely to VA were included in molecular docking. The docking information of components and hub targets was listed in Table 4, and the results suggested that components in DFD could interact with the hub targets against VA, following the principle that the lower the binding energy, the more stable the docking modules [45]. The lowest 10 binding energy docking modules are shown in Figure 6, the rest docking module could be found in the supplement, and all 10 components interacted with corresponding targets through hydrogen bond mainly.

The results show that Jujubogenin has the highest binding energy connected with HIS70 and ASP67 of KCNH2 (Figure 6(a)), acacic acid lactone connected with ARG62 of KCNH2 (Figure 6(b)), kanzonols W connected with ASP88 of TNNT2 (Figure 6(c)), stepharine connected with LEU86 of KCNH2 (Figure 6(d)), N-methylasimilobine connected with HIS70 of KCNH2 (Figure 6(e)), DFV connected with ARG86 and ARG90 of CALM1 (Figure 6(f)), asimilobine connected with LYS101 of KCNH2 (Figure 6(g)), cadaverine connected with VAL36 of KCNH2 (Figure 6(h)), nuciferine connected with GLU95 of KCNH2 (Figure 6(i)), and 7-acetoxy-2-methylisoflavone connected with LYS93 of KCNH2 (Figure 6(j)).

## 4. Discussion

VA is a fatal disease, typical drugs may benefit patients, but its side effects such as respiratory diseases, liver and kidney damage, and bradyarrhythmia can never be ignored. Fortunately, long-time clinical work was told that DFD is an effective herb mixture against antiarrhythmia. Since its excellent clinical efficacy, we conducted a Real-World Trial that included more than 160 patients who suffered premature ventricular contractions to assess the safety and efficacy of DFD for VA and the results demonstrate that DFD combined with metoprolol has better efficacy and safety than placebo combined with metoprolol [24]. Besides, we explored the cellular electrophysiological mechanism of DFD with Chinese hamster ovary cells using whole-cell patch-clamp, and the result suggests that DFD indeed has antiarrhythmic effects based on its antioxidant potential, alleviation of Na^+^-K^+^-ATPase and connexin-43, and class I antiarrhythmic properties by suppressing Nav_1.5_ dose-dependently with an IC_50_ of 24.0 ± 2.4 mg/mL [25]. In this study, the bioactive components and underlying molecular mechanisms of DFD in the treatment of VA were analyzed systematically.

Through related information collection and primary screening, we identified 28 potential targets of DFD in the treatment of VA. A PPI network was constructed with STRING and Cytoscape 3.8.0, the top 10° value genes were selected as hub genes, and 3 function modules were divided based on their interactions. All potential genes were analyzed using VarElect, all 10 hub genes are suggested directly related to the treatment of VA, and among these genes, KCNH2, TNNT2, and CALM1 as well as SCN5A have the highest scores of correlations; in other words, these 4 genes are the most promising targets for DFD against VA. Recently, KCNH2 could be a hot gene in the study of VA; it could mediate the rapidly activating component of the delayed rectifying potassium current in the heart. Previous research suggested that pathogenic variants in KCNH2 encoding may result in long QT syndrome [46]. Meanwhile, another research based on quantitative analysis of consortium disease cohorts and population controls pointed out that, among patients with long QT syndrome, the mutation probability of the KCNH2 gene is greater than 85% [47]. Besides, another research mentioned the coexpression of CACNA1C and KCNH2 reduces the arrhythmic events [48]. TNNT2 is another hub gene connected to arrhythmias; a genetic analysis suggested that TNNT2 was cosegregated in VAs and sudden death [49]. A study conducted using zebrafish embryos suggested that zebrafish embryos exposed to procymidone are more likely to alter transcription levels of TNNT2 and resulted in arrhythmia as well as increased heart rate finally [50]. Raffaele Coppini conducted a cohort study of patients with hypertrophic cardiomyopathy (HCM), the outcome indicated that, among patients with HCM, most patients have a mutation in TNNT2, and these patients are more likely to suffer from arrhythmias and HCM in the future [51]. SCN5A is pivotal to cardiac electrical conduction and arrhythmic risk; a study provided a new effective therapy to reduce arrhythmia through downregulating the expression of SCN5A [52]. Similarly, there is a study that reported that a combination of quinidine/mexiletine reduces arrhythmia in patients with SCN5A gene mutation [53]. CALM1 is a regulator of voltage-dependent L-type calcium channels; its mutations are related to congenital arrhythmia [54]. Heterozygosity for the CALM1 mutation is causative of an arrhythmia syndrome [55]. Moreover, it can lead to catecholaminergic polymorphic ventricular tachycardia, idiopathic ventricular fibrillation, long QT syndrome, and even sudden death [56].

In the further, the results of GO and KEGG analysis elucidated that the regulation of systemic arterial blood pressure by norepinephrine-epinephrine, muscle contraction, blood circulation, circulatory system process, adrenergic receptor activity, calcium signaling pathway, adenylate signaling in cardiomyocytes, cGMP-PKG signaling pathway, and neuroactive ligand-receptor interaction were highly enriched, which revealed the potential mechanisms of DFD in treating VA. Here, the regulation of the calcium signaling pathway is enriched by most of the hub genes. According to a study based on the genomic, transcriptomic, and proteomic data initiated by Dan E Arking, calcium signaling pathway plays an important role in both the depolarization and repolarization of myocardial ischemia; particularly in the repolarization, during the plateau phase of the cardiac action potential, prolonged inward Ca2+ currently leads to delays in ventricular myocyte repolarization [57]. Earlier research also mentioned that Ca^2+^ waves can form when the Ca^2+^ ion influx into the cell is increased, and the Ca^2+^ waves can generate depolarization that triggers arrhythmias [58], it is reasonable to speculate that the regulation of calcium signaling pathways of DFD may be one of the effective methods for VA. Reports also suggested that the adrenergic signaling can increase the transmural difference between Ca^2+^ ion transients duration and action potential duration, finally, promoting the formation of delayed afterdepolarizations, the regulation of adenylate cyclase-activating adrenergic receptor signaling pathway and adrenergic receptor signaling pathway of DFD for VA may antiarrhythmia in this way [59]. Adenylate cyclase-modulating G protein-coupled receptor signaling pathway can result in the regulation of G protein-mediated signaling, which is of great importance for the regulation of heart rate and involved in arrhythmias [60]. Besides, as we mentioned above, the potential targets were divided into 3 function modules, as shown in Table 3. The enrichment analysis results indicate Module 1 can regulate calcium signaling pathway, heart rate, and cAMP signaling pathway, which suggest that Module 1 has great potential in the antiarrhythmia. Similarly, the results of enrichment analysis indicate that Module 2 were involved in the regulation of membrane depolarization during an action potential, striated muscle contraction, regulate adrenergic signaling in cardiomyocytes and cation homeostasis, pathways, which has illustrated the antiarrhythmic potential of Module 2. The enrichment analysis of Module 3 may not seem to be ideal, but we found that the 4 genes connected to VA closest are gathered in function Module 3, and it is reasonable to believe that Module 3 has antiarrhythmic effects. Furthermore, as is shown in Figure 5, the multiregulation in different aspects may benefit patients suffering from related diseases such as hypertension, cancer, and other diseases.

Nevertheless, the present study has some limitations. First, although we tried to find out all components, *Osdraconis* (*Fossiliaossiamastodi*) and *Ostrea Gigas Thunberg,* has only several pieces of research with several components, and were excluded for its poor possibility of GA and DL, but *Osdraconis* (*Fossiliaossiamastodi*) and *Ostrea Gigas Thunberg* played important roles in DFD; according to the theoretical system of TCM, both *Osdraconis* (*Fossiliaossiamastodi*) and *Ostrea Gigas Thunberg* can tranquilize the mind; further studies are needed to confirm the sedative mechanism of *Osdraconis* (*Fossiliaossiamastodi*) and *Ostrea Gigas Thunberg*. Moreover, DFD, as a traditional Chinese decoction, treats VA through multicomponent and multitarget, indicating that the underlying mechanisms are complex as described in our present study. Although we have made some identification on the mechanisms [25], there are still a lot to explore. Here, this study is designed to understand the mechanism profoundly through network pharmacology as well as molecular docking technology. Same as other network pharmacology analyses [37], taking the intersection of targets about DFD and VA in this study not only follows the operating processes of network pharmacology but also makes the result of virtual screening more reliable [61]. Undoubtedly, some targets that involve either DFD or VA might be ignored and missed, which is a common and inevitable issue in network pharmacology. And for this reason, in the collection of components and targets, despite DFD and VA, we tried our best to reduce bias by searching as much more databases as we can. Finally, so many components are boiled together, and more researches are needed to detect whether there are some new formed compounds.

## 5. Conclusion

As mentioned above, DFD could be employed for VA through mechanisms, including complex interactions between related components and targets, as predicted by network pharmacology and molecular docking. This work confirmed that DFD could apply to the treatment of VA and promotes the explain of DFD for VA in the molecular mechanisms; similar results can be obtained from previous experiments of cellular electrophysiological mechanisms. The systematic analysis in this work can provide a comprehensive consideration for further studies.

## Figures and Tables

**Table 1:** Designations and topological parameters of hub genes in the PPI network.

Gene symbol	Protein name	Degree	Betweenness centrality	Closeness centrality
KCNH2	Potassium voltage-gated channel subfamily H member 2	10	128.5	0.32
CYP3A4	Cytochrome P450 3A4	9	166.8	0.35
CYP2C9	Cytochrome P450 2C9	8	22.2	0.31
PIK3R1	Phosphatidylinositol 3-kinase regulatory subunit alpha	8	146.7	0.31
CHRM1	Muscarinic acetylcholine receptor M1	7	19.2	0.27
CYP2D6	Cytochrome P450 2D6	7	59.1	0.32
EGFR	Epidermal growth factor receptor	7	228.3	0.36
SLCO1B1	Solute carrier organic anion transporter family member 1B1	6	3.9	0.28
ADRA1B	Alpha-1B adrenergic receptor	6	11.1	0.27
ADRB2	Beta-2 adrenergic receptor	6	63.8	0.31

**Table 2:** The connection information of shared genes.

Genes	Description	Relationship	Score
SCN5A	Sodium voltage-gated channel alpha subunit 5	Directly	445.8
KCNH2	Potassium voltage-gated channel subfamily H member 2	Directly	409.76
TNNT2	Troponin T2, cardiac type	Directly	293.82
CALM1	Calmodulin 1	Directly	222.12
SCN4A	Sodium voltage-gated channel alpha subunit 4	Directly	158.41
SCN10 A	Sodium voltage-gated channel alpha subunit 10	Directly	132.53
ADRB1	Adrenoceptor beta 1	Directly	97.00
ADRB2	Adrenoceptor beta 2	Directly	75.14
CYP2C9	Cytochrome P450 family 2 subfamily C member 9	Directly	53.13
PDGFRB	Platelet-derived growth factor receptor beta	Directly	46.86
CHRM2	Cholinergic receptor muscarinic 2	Directly	46.86
MAPK1	Mitogen-activated protein kinase 1	Directly	43.38
EGFR	Epidermal growth factor receptor	Directly	43.38
ADRA1D	Adrenoceptor alpha 1D	Directly	39.60
ADRB3	Adrenoceptor beta 3	Directly	39.60
SCN9A	Sodium voltage-gated channel alpha subunit 9	Directly	35.42
CYP3A4	Cytochrome P450 family 3 subfamily A member 4	Directly	30.68
CYP2D6	Cytochrome P450 family 2 subfamily D member 6	Directly	25.05
PON1	Paraoxonase 1	Directly	25.05
PIK3R1	Phosphoinositide-3-kinase regulatory subunit 1	Directly	17.71
CYP1A1	Cytochrome P450 family 1 subfamily A member 1	Directly	17.71
CHRM1	Cholinergic receptor muscarinic 1	Directly	17.71
CYP1A2	Cytochrome P450 family 1 subfamily A member 2	Directly	17.71
ADRA1A	Alpha-1A adrenergic receptor	Indirectly	37.78
ADRA1B	Alpha-1B adrenergic receptor	Indirectly	36.23
CHRM3	Muscarinic acetylcholine receptor M3	Indirectly	28.09
SLCO1B1	Solute carrier organic anion transporter family member 1B1	Indirectly	13.50
SLC47A1	Multidrug and toxin extrusion protein 1	Indirectly	11.33

Notes: the score is an indication of the strength of the connection between the gene and the disease.

**Table 3:** GO and KEGG enrichment analysis of the biofunction modules.

Function modules	Description	Log_10_ (*P* value)
Module 1	ko04020, calcium signaling pathway	−22.3027025
	GO:0001996, positive regulation of heart rate by epinephrine-norepinephrine	−12.3777817
	ko04810, regulation of actin cytoskeleton	−11.6704785
	GO:0043410, positive regulation of MAPK cascade	−10.5659921
	ko04024, cAMP signaling pathway	−9.71878217

Module 2	GO:0086010, membrane depolarization during action potential	−12.7696584
	GO:0006941, striated muscle contraction	−11.8188992
	ko04261, adrenergic signaling in cardiomyocytes	−7.33482642
	GO:0019233, sensory perception of pain	−5.53543107
	GO:0055080, cation homeostasis	−3.03192747

Module 3	GO:0016098, monoterpenoid metabolic process	−13.3868655
	GO:0008202, steroid metabolic process	−10.1366162
	GO:0006690, icosanoid metabolic process	−10.0957724
	GO:0008203, cholesterol metabolic process	−9.58364991
	GO:0010035, response to inorganic substance	−3.36527057

**Table 4:** The related information of components docked with key targets.

Gene	Component	PubChem CLD	Origin	Binding energy (kcal/mol)
KCNH2	Jujubogenin	15515703	Semen	−4.85
KCNH2	Acacic acid lactone	6712546	Silktree Albizia Bark	−4.83
KCNH2	Stepharine	98455	Jujubae Fructus	−4.52
KCNH2	N-Methylasimilobine	197017	Semen	−4.29
KCNH2	Asimilobine	160875	Jujubae Fructus	−4.2
KCNH2	Cadaverine	23335	Semen	−4.16
KCNH2	Nuciferine	10146	Jujubae Fructus	−4.07
KCNH2	7-Acetoxy-2-methylisoflavone	268208	Licorice	−4.02
KCNH2	(S)-Coclaurine	160487	Semen; Jujubae Fructus	−3.42
KCNH2	Juzirine	3085285	Semen	−3.02
KCNH2	(2S)-6-(2,4-Dihydroxyphenyl)-2-(2-hydroxypropan-2-yl)-4-methoxy-2,3-dihydrofuro[3,2-g]chromen-7-one	637112	Licorice	−2.96
KCNH2	Zizyphusine	102063083	Semen	−2.8
KCNH2	Senkyunone	91726743	Chuanxiong Rhizoma	−2.59
KCNH2	25-Hydroxy-3-epidehydrotumulosic acid	10368709	*Poria cocos* (Schw.)	−2.09
KCNH2	Ethyl pentadecanoate	38762	Chuanxiong Rhizoma	−0.61
KCNH2	AP1	21119850	*Silktree Albizia Bark*	1.45
TNNT2	Kanzonols W	15380912	Licorice	−4.62
TNNT2	Glabridin	124052	Licorice	−4.02
TNNT2	Senkyunolide G	5321250	Chuanxiong Rhizoma	−3.23
TNNT2	Odoratin	13965473	Licorice	−3.05
CALM1	DFV	114829	Licorice	−4.21
CALM1	Licochalcone B	5318999	Licorice	−2.66
SCN5A	Senkyunone	91726743	Chuanxiong Rhizoma	−1.59

The binding energy refers to the strength of the binding between the receptor and the ligand; the lower the binding energy, the more stable the docking module.

## Data Availability

All the data generated or analyzed during this study are included in this published article and its supplementary information files.

## Abbreviations

DFD: Dingji Fumai Decoction
VA: Ventricular arrhythmia
TCM: Traditional Chinese medicine
GA: Gastrointestinal absorption
DL: Drug-likeness
H-C-T-D: Herb-compound-target-disease
TCMSP: Traditional Chinese Medicine Systems Pharmacology database and analysis platform
ETCM: Encyclopedia of Traditional Chinese Medicine
ADME: Absorption, distribution, metabolism, and excretion
PPI: Protein-protein interaction
GO: Gene ontology
KEGG: Kyoto encyclopedia of genes and genomes
KCNH2: Potassium voltage-gated channel subfamily H member 2
SCN5A: Sodium voltage-gated channel alpha subunit 5
TNNT2: Troponin T2-cardiac type
CALM1: Calmodulin 1
HCM: Hypertrophic cardiomyopathy.

## Supplementary Materials

Supplementary Materials Table S1: comparison of Chinese medicine names and Latin names. Table S2: details of qualified compounds in various herbs. Figure S1: molecular docking modules.
