# Supplementary material for: Network Pharmacology-Based Systematic Analysis of Molecular Mechanisms of Dingji Fumai Decoction for Ventricular Arrhythmia
Source: Evid Based Complement Alternat Med. 2021 May 8;2021:5535480. doi: 10.1155/2021/5535480 (PMC8128550; doi:10.1155/2021/5535480)
Supplement: Supplementary Materials — Table S1: comparison of Chinese medicine names and Latin names. Table S2: details of qualified compounds in various herbs. Figure S1: molecular docking modules. [file 5535480.f1.zip › 5535480.f1/Figure S1. Molecular docking modules.pdf]

Figure S1. Molecular docking modules.

CALM1

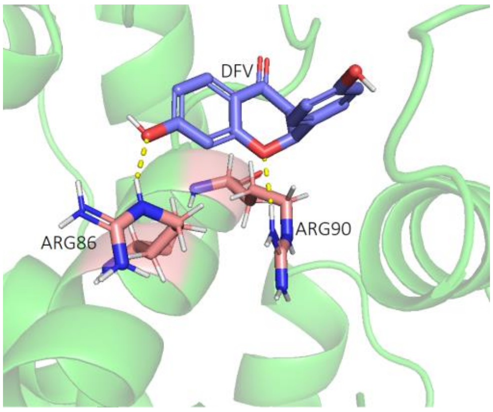

DFV- CALM1

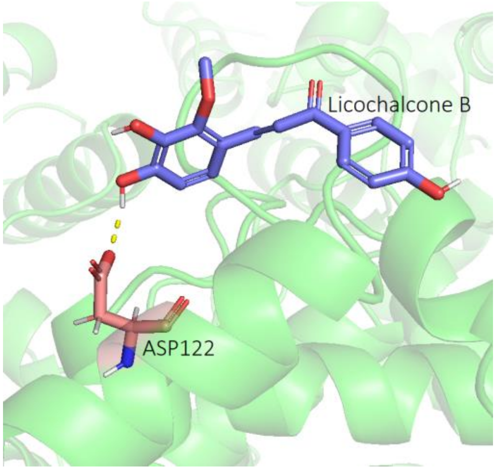

Licochalcone B- CALM1

KCNH2

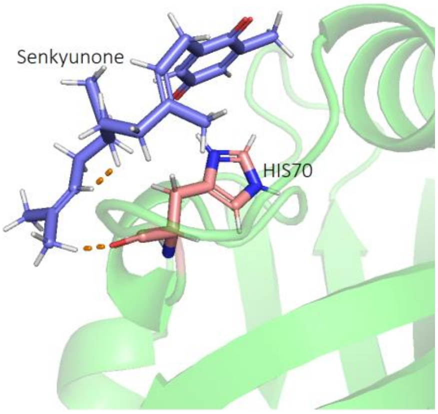

Senkyunone-KCNH2

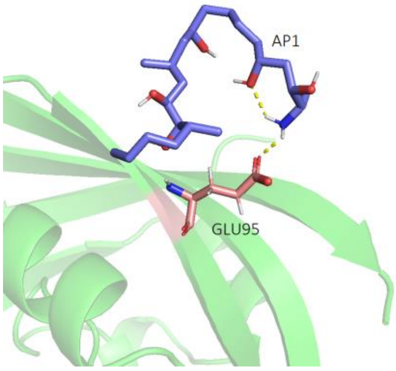

AP1- KCNH2

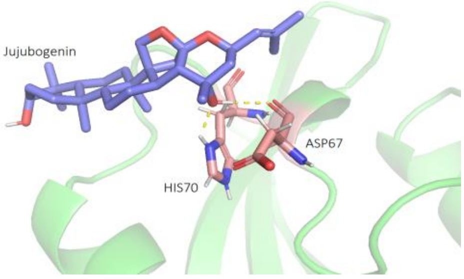

Jujubogenin-KCNH2

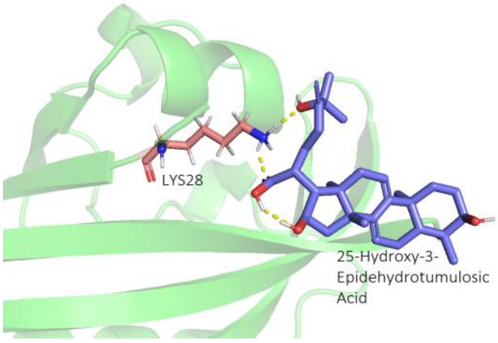

25-Hydroxy-3-Epidehyrotumulosic Acid

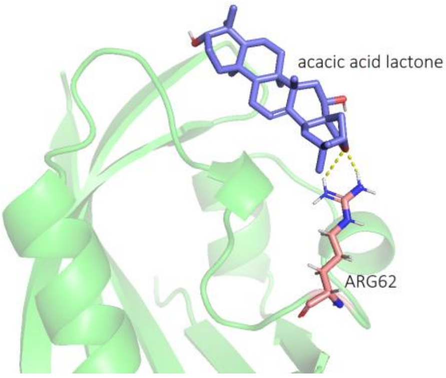

Acacic acid lactone-KCNH2

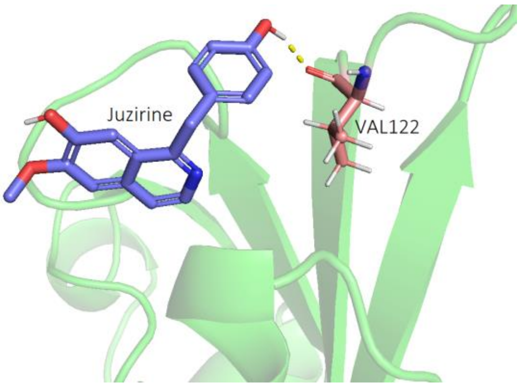

Juzirine-KCNH2

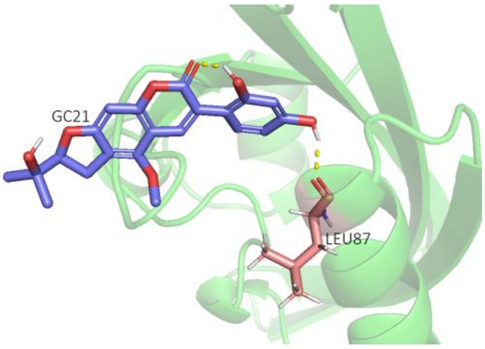

GC21-KCNH2

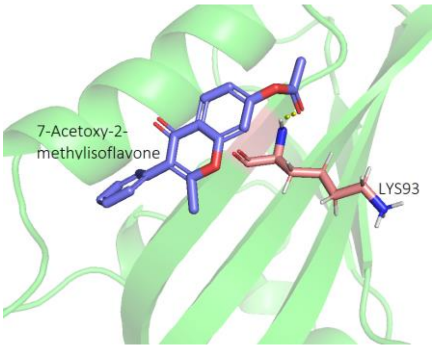

7-Acetoxy-2-methvlisoflavone-KCNH2

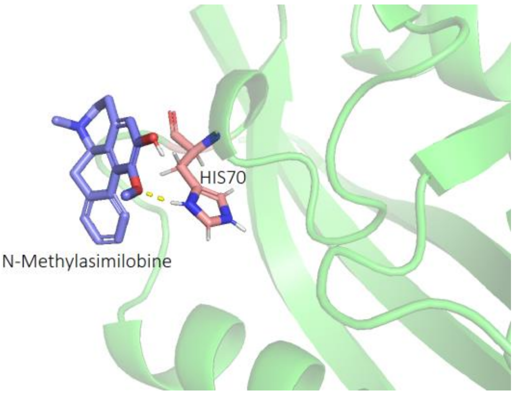

N-Methylasimilobine-KCNH2

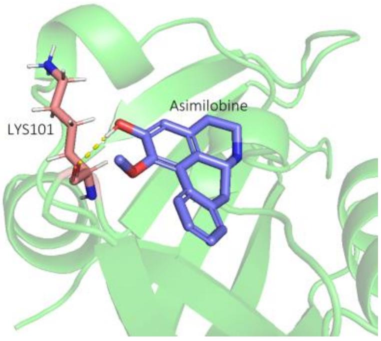

Asimilobine-KCNH2

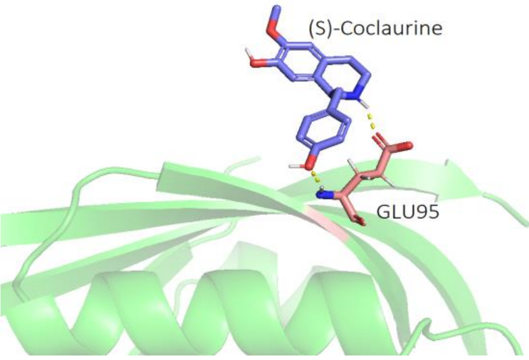

(S)-Coclaurine-KCNH2

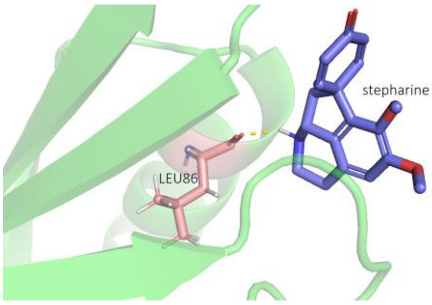

Stepharine-KCNH2

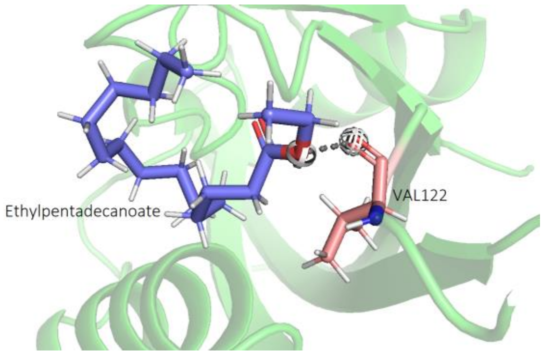

Ethylpentadecanoate-KCNH2

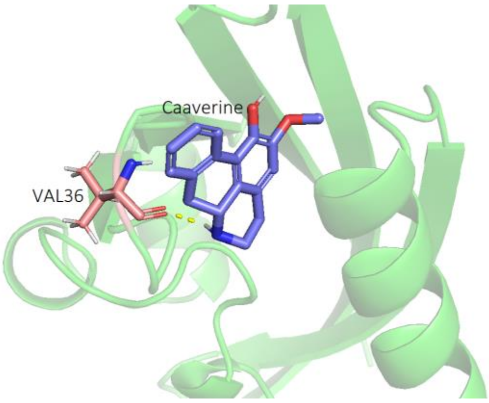

Caaverine-KCNH2

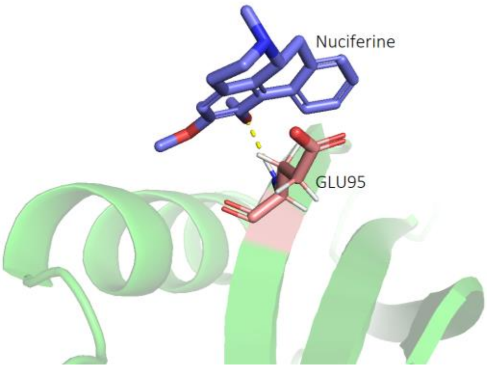

Nuciferine-KCNH2

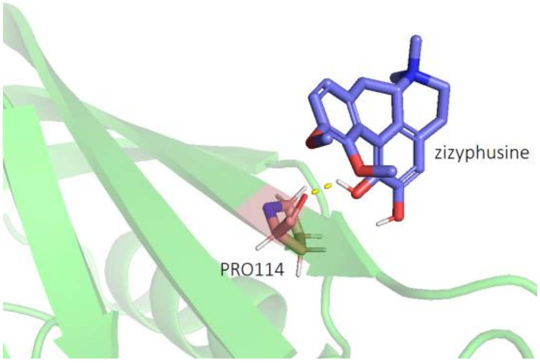

Zizyphusine-KCNH2

SCN5A

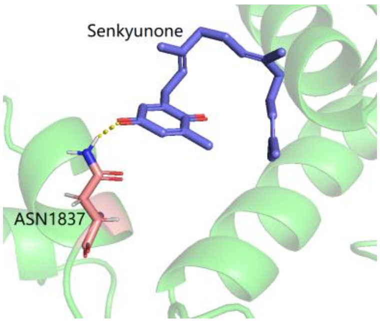

Senkyunone-SCN5A

TNNT2

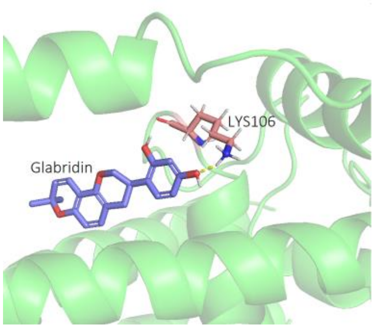

Glabridin-TNNT2

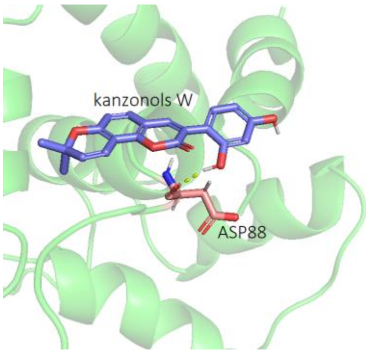

kanzonols W-TNNT2

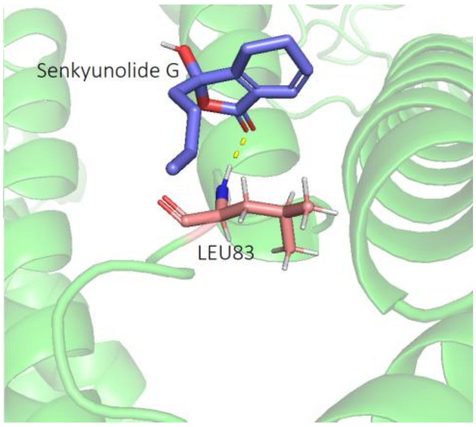

Senkyunolide G-TNNT2

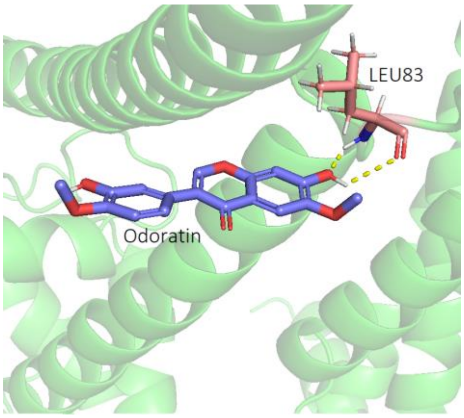

Odoratin-TNNT2
