# Supplementary material for: Network Pharmacology-Based Systematic Analysis of Molecular Mechanisms of Dingji Fumai Decoction for Ventricular Arrhythmia
Source: Evid Based Complement Alternat Med. 2021 May 8;2021:5535480. doi: 10.1155/2021/5535480 (PMC8128550; doi:10.1155/2021/5535480)
Supplement: Supplementary Materials — Table S1: comparison of Chinese medicine names and Latin names. Table S2: details of qualified compounds in various herbs. Figure S1: molecular docking modules. [file 5535480.f1.zip › 5535480.f1/Table S2.docx]

### Table 1s . Details of qualified compounds in various herbs.

| PUBCHEM CLD | ingredients | SwissADME results | | | | | |  |
| --- | --- | --- | --- | --- | --- | --- | --- | --- |
|  |  | Gatrointestinal absorption(GA) | druglikeness (DL) | | | | |  |
|  |  |  | lipinski | ghose | veber | egan | muegge | |
| 5280343 | Quercetin | high | √ | √ | √ | √ | √ | |
| 72 | Protocatechuic Acid | high | √ |  | √ | √ |  | |
| 985 | Cetylic Acid | high | √ | √ |  | √ |  | |
| 689043 | Caffeic Acid | high | √ | √ | √ | √ |  | |
| 5321250 | Senkyunolide G | high | √ | √ | √ | √ | √ | |
| 15138552 | Senkyunolide N | high | √ | √ | √ | √ | √ | |
| 91726743 | Senkyunone | high | √ |  | √ | √ |  | |
| 92231 | Spathulenol | high | √ | √ | √ | √ |  | |
| 1174 | Uracil | high | √ |  | √ | √ |  | |
| 1183 | Vanillin | high | √ |  | √ | √ |  | |
| 122736 | Wallichilide | high | √ |  | √ | √ | √ | |
| 522266 | Spathulenol | high | √ | √ | √ | √ |  | |
| 10288 | Chrysophanic Acid | high | √ | √ | √ | √ | √ | |
| 10208 | Chrysophanol | high | √ | √ | √ | √ | √ | |
| 14900 | Dilinoyl Palmitoyl Glyceride,Glycerol1-Monopalmitate | high | √ | √ |  | √ |  | |
| 38762 | Ethylpentadecanoate | high | √ |  |  | √ |  | |
| 8181 | Methyl Hexadecanate | high | √ |  |  | √ |  | |
| 23518 | Methyl Pentadecanoate | high | √ | √ |  | √ |  | |
| 161748 | Myricanone | high | √ | √ | √ | √ | √ | |
| 445354 | Retinol | high | √ | √ | √ | √ |  | |
| 445858 | 3-O-trans ferulylquinic acid | high | √ | √ | √ | √ |  | |
| 98455 | stepharine | high | √ | √ | √ | √ | √ | |
| 5280450 | Linoleic Acid | high | √ |  | √ |  |  | |
| 11005 | Myristic Acid | high | √ | √ |  | √ |  | |
| 5281 | Stearic Acid | high | √ |  | √ |  |  | |
| 222656 | Malic Acid | high | √ |  | √ | √ |  | |
| 938 | Nicotinic Acid | high | √ |  | √ | √ |  | |
| 6267 | Asparagine | high | √ |  | √ | √ |  | |
| 470606 | Alphitolic Acid | high | √ |  | √ |  |  | |
| 445638 | Palmitoleic Acid | high | √ | √ |  | √ |  | |
| 160875 | Asimilobine | high | √ | √ | √ | √ | √ | |
| 289 | Catechol | high | √ |  | √ | √ |  | |
| 5281707 | coumestrol | high | √ | √ | √ | √ | √ | |
| 21672700 | Colubrinic Acid | high | √ |  | √ |  |  | |
| 73659 | 2Î‘,3Î’-Dihydroxyolean-12-En-28-Oic Acid | high | √ |  | √ |  |  | |
| 1130 | Vitamin B1 | high | √ | √ | √ | √ | √ | |
| 5280462 | Vomifoliol | high | √ | √ | √ | √ | √ | |
| 6443026 | Mauritine D | high | √ |  |  | √ |  | |
| 2353 | berberine | high | √ | √ | √ | √ | √ | |
| 101650325 | Ruvoside | high | √ |  | √ |  | √ | |
| 6917970 | Stepholidine | high | √ | √ | √ | √ | √ | |
| 10146 | Nuciferine | high | √ | √ | √ | √ | √ | |
| 4970 | Fumarine | high | √ | √ | √ | √ | √ | |
| 5280537 | Moupinamide | high | √ | √ | √ | √ | √ | |
| 5351516 | Peroxyergosterol | high | √ |  | √ |  |  | |
| 160487 | (S)-Coclaurine | high | √ | √ | √ | √ | √ | |
| 122691 | Lysicamine | high | √ | √ | √ | √ |  | |
| 73299 | hederagenin | high | √ |  | √ |  |  | |
| 10181133 | Cerevisterol | high | √ |  | √ | √ |  | |
| 10743008 | (2R)-2-[(3S,5R,10S,13R,14R,16R,17R)-3,16-dihydroxy-4,4,10,13,14-pentamethyl-2,3,5,6,12,15,16,17-octahydro-1H-cyclopenta[a]phenanthren-17-yl]-6-methylhept-5-enoic acid | high | √ |  | √ |  |  | |
| 3893 | Lauric Acid | high | √ | √ | √ | √ | √ | |
| 379 | Caprylic Acid | high | √ |  | √ | √ |  | |
| 125207 | Dodecenoic Acid | high | √ | √ | √ | √ | √ | |
| 190 | Adenine | high | √ |  | √ | √ |  | |
| 10368709 | 25-Hydroxy-3-Epidehydrotumulosic Acid | high | √ |  | √ | √ | √ | |
| 8180 | Undecanoic Acid | high | √ | √ | √ | √ |  | |
| 91510 | Inermine | high | √ | √ | √ | √ | √ | |
| 5280448 | Calycosin | high | √ | √ | √ | √ | √ | |
| 5280863 | kaempferol | high | √ | √ | √ | √ | √ | |
| 439246 | naringenin | high | √ | √ | √ | √ | √ | |
| 197678 | (2S)-2-[4-hydroxy-3-(3-methylbut-2-enyl)phenyl]-8,8-dimethyl-2,3-dihydropyrano[2,3-f]chromen-4-one | high | √ | √ | √ | √ |  | |
| 10291003 | euchrenone | high | √ | √ | √ | √ | √ | |
| 480784 | glyasperin B | high | √ | √ | √ | √ | √ | |
| 392442 | glyasperin F | high | √ | √ | √ | √ | √ | |
| 480859 | Glyasperin C | high | √ | √ | √ | √ | √ | |
| 5318679 | Isotrifoliol | high | √ | √ | √ | √ | √ | |
| 10881804 | (E)-1-(2,4-dihydroxyphenyl)-3-(2,2-dimethylchromen-6-yl)prop-2-en-1-one | high | √ | √ | √ | √ | √ | |
| 114829 | DFV | high | √ | √ | √ | √ | √ | |
| 15380912 | kanzonols W | high | √ | √ | √ | √ | √ | |
| 637112 | (2S)-6-(2,4-dihydroxyphenyl)-2-(2-hydroxypropan-2-yl)-4-methoxy-2,3-dihydrofuro[3,2-g]chromen-7-one | high | √ | √ | √ | √ | √ | |
| 5481948 | Semilicoisoflavone B | high | √ | √ | √ | √ | √ | |
| 5281619 | Glepidotin A | high | √ | √ | √ | √ | √ | |
| 442411 | Glepidotin B | high | √ | √ | √ | √ | √ | |
| 162412 | Phaseolinisoflavan | high | √ | √ | √ | √ | √ | |
| 5317768 | Glypallichalcone | high | √ | √ | √ | √ | √ | |
| 10542808 | 8-(6-hydroxy-2-benzofuranyl)-2,2-dimethyl-5-chromenol | high | √ | √ | √ | √ | √ | |
| 5318999 | Licochalcone B | high | √ | √ | √ | √ | √ | |
| 49856081 | licochalcone G | high | √ | √ | √ | √ |  | |
| 5320083 | Glycyrol | high | √ | √ | √ | √ | √ | |
| 10090416 | 3-(2,4-dihydroxyphenyl)-8-(1,1-dimethylprop-2-enyl)-7-hydroxy-5-methoxy-coumarin | high | √ | √ | √ | √ | √ | |
| 5319013 | Licoricone | high | √ | √ | √ | √ | √ | |
| 5317478 | Gancaonin A | high | √ | √ | √ | √ | √ | |
| 5317479 | Gancaonin B | high | √ | √ | √ | √ | √ | |
| 14604077 | 3-(3,4-dihydroxyphenyl)-5,7-dihydroxy-8-(3-methylbut-2-enyl)chromone | high | √ | √ | √ | √ | √ | |
| 14604078 | 5,7-dihydroxy-3-(4-methoxyphenyl)-8-(3-methylbut-2-enyl)chromone | high | √ | √ | √ | √ | √ | |
| 14604081 | 2-(3,4-dihydroxyphenyl)-5,7-dihydroxy-6-(3-methylbut-2-enyl)chromone | high | √ | √ | √ | √ | √ | |
| 480787 | Glycyrin | high | √ | √ | √ | √ | √ | |
| 503731 | Licocoumarone | high | √ | √ | √ | √ | √ | |
| 5281789 | Licoisoflavone | high | √ | √ | √ | √ | √ | |
| 5318869 | Jaranol | high | √ | √ | √ | √ | √ | |
| 5481234 | Licoisoflavone B | high | √ | √ | √ | √ | √ | |
| 392443 | licoisoflavanone | high | √ | √ | √ | √ | √ | |
| 10336244 | shinpterocarpin | high | √ | √ | √ | √ | √ | |
| 11267805 | (E)-3-[3,4-dihydroxy-5-(3-methylbut-2-enyl)phenyl]-1-(2,4-dihydroxyphenyl)prop-2-en-1-one | high | √ | √ | √ | √ | √ | |
| 122851 | licopyranocoumarin | high | √ | √ | √ | √ | √ | |
| 195396 | 3,22-Dihydroxy-11-oxo-delta(12)-oleanene-27-alpha-methoxycarbonyl-29-oic acid | high | √ |  | √ | √ | √ | |
| 5317777 | Glyzaglabrin | high | √ | √ | √ | √ | √ | |
| 124052 | Glabridin | high | √ | √ | √ | √ | √ | |
| 124049 | Glabranin | high | √ | √ | √ | √ | √ | |
| 480774 | Glabrene | high | √ | √ | √ | √ | √ | |
| 336327 | Medicarpin | high | √ | √ | √ | √ | √ | |
| 5317652 | Glabrone | high | √ | √ | √ | √ | √ | |
| 11558452 | 1,3-dihydroxy-9-methoxy-6-benzofurano[3,2-c]chromenone | high | √ | √ | √ | √ | √ | |
| 11602329 | 1,3-dihydroxy-8,9-dimethoxy-6-benzofurano[3,2-c]chromenone | high | √ | √ | √ | √ | √ | |
| 5317300 | Eurycarpin A | high | √ | √ | √ | √ | √ | |
| 23724664 | (-)-Medicocarpin | high | √ | √ | √ | √ | √ | |
| 73205 | Sigmoidin-B | high | √ | √ | √ | √ | √ | |
| 928837 | (2R)-7-hydroxy-2-(4-hydroxyphenyl)chroman-4-one | high | √ | √ | √ | √ | √ | |
| 193679 | (2S)-7-hydroxy-2-(4-hydroxyphenyl)-8-(3-methylbut-2-enyl)chroman-4-one | high | √ | √ | √ | √ | √ | |
| 124050 | Isoglycyrol | high | √ | √ | √ | √ | √ | |
| 5318585 | Isolicoflavonol | high | √ | √ | √ | √ | √ | |
| 5281654 | isorhamnetin | high | √ | √ | √ | √ | √ | |
| 3764 | HMO | high | √ | √ | √ | √ | √ | |
| 480873 | 1-Methoxyphaseollidin | high | √ | √ | √ | √ | √ | |
| 5316900 | Quercetin der. | high | √ | √ | √ | √ | √ | |
| 15228662 | 3'-Hydroxy-4'-O-Methylglabridin | high | √ | √ | √ | √ | √ | |
| 5318998 | licochalcone a | high | √ | √ | √ | √ | √ | |
| 15228663 | 3'-Methoxyglabridin | high | √ | √ | √ | √ | √ | |
| 9927807 | 2-[(3R)-8,8-dimethyl-3,4-dihydro-2H-pyrano[6,5-f]chromen-3-yl]-5-methoxyphenol | high | √ | √ | √ | √ | √ | |
| 5318437 | Inflacoumarin A | high | √ | √ | √ | √ | √ | |
| 101666840 | Kanzonol F | high | √ | √ | √ | √ |  | |
| 25015742 | 7,2',4'-trihydroxy－5-methoxy-3－arylcoumarin | high | √ | √ | √ | √ | √ | |
| 5317480 | Lupiwighteone | high | √ | √ | √ | √ | √ | |
| 268208 | 7-Acetoxy-2-methylisoflavone | high | √ | √ | √ | √ | √ | |
| 177149 | Vestitol | high | √ | √ | √ | √ | √ | |
| 480780 | Gancaonin G | high | √ | √ | √ | √ | √ | |
| 5481949 | Gancaonin H | high | √ | √ | √ | √ |  | |
| 15840593 | Licoagrocarpin | high | √ | √ | √ | √ | √ | |
| 5317765 | Glycyrrhiza flavonol A | high | √ | √ | √ | √ | √ | |
| 636883 | Licoagroisoflavone | high | √ | √ | √ | √ | √ | |
| 13965473 | Odoratin | high | √ | √ | √ | √ | √ | |
| 44257530 | Phaseol | high | √ | √ | √ | √ | √ | |
| 14769500 | Xambioona | high | √ | √ | √ | √ | √ | |
| 354368 | 7-Methoxy-2-methyl isoflavone | high | √ | √ | √ | √ | √ | |
| 5280378 | formononetin | high | √ | √ | √ | √ | √ | |
| 323 | Coumarin | high | √ |  | √ | √ |  | |
| 444539 | Cinnamic Acid | high | √ |  | √ | √ |  | |
| 11604108 | (-)-Syringaresinol | High | √ | √ | √ | √ | √ | |
| 614467 | 5-(2-Methyl-1,3-thiazol-4-yl)thiophene-2-carboxylic acid | high | √ | √ | √ | √ | √ | |
| 2794766 | 2-[4-(Trifluoromethyl)phenyl]-1,3-thiazole-4-carboxylic Acid | high | √ | √ | √ | √ | √ | |
| 135567045 | 2-(4-((Pyridin-4-Yl)methyl)piperazin-1-Yl)-3,4,5,6,7,8-Hexahydroquinazolin-4-One | high | √ | √ | √ | √ | √ | |
| 196916 | Julibrine Ⅰ | high | √ | √ | √ |  |  | |
| 21594250 | Macharinic acid lactone | high | √ | √ | √ | √ | √ | |
| 5281646 | Macluraxanthone | high | √ | √ | √ | √ |  | |
| 69997336 | Norarmepavine | high | √ | √ | √ | √ | √ | |
| 54670067 | Vitamin C | high | √ |  | √ | √ |  | |
| 9064 | D-Catechin | high | √ | √ | √ | √ | √ | |
| 12305894 | acacic acid | high | √ |  | √ | √ |  | |
| 6712546 | acacic acid lactone | high | √ |  | √ | √ |  | |
| 21119850 | AP1 | high | √ |  |  | √ |  | |
| 444664 | AP3 | high | √ | √ | √ |  |  | |
| 73309 | Echinocystic acid | high | √ |  | √ |  |  | |
| 725031 | 2-[(4-Methylphenyl)thio]nicotinic acid | high | √ | √ | √ | √ | √ | |
| 11453158 | Keratinocyte Differentiation Inducer | high | √ | √ | √ | √ | √ | |
| 131990 | 10-(4-Methylpiperazin-1-yl)pyrido(4,3-b)(1,4)benzothiazepine | high | √ | √ | √ | √ | √ | |
| 33032 | L-glutamic acid | high | √ |  | √ | √ |  | |
| 5960 | l-aspartic acid | high | √ |  | √ | √ |  | |
| 5962 | lysine | high | √ |  | √ | √ |  | |
| 205 | DL-Threonine | high | √ |  | √ | √ |  | |
| 14729078 | sanjoinenine | hgih | √ |  | √ | √ |  | |
| 102063083 | zizyphusine | hgih | √ | √ | √ | √ |  | |
| 12305768 | Alphitolic Acid | high | √ |  | √ |  |  | |
| 197017 | N-Methylasimilobine | high | √ | √ | √ | √ |  | |
| 23335 | Caaverine | high | √ | √ | √ | √ |  | |
| 15515703 | Jujubogenin | high | √ |  | √ | √ |  | |
| 3085285 | Juzirine | high | √ | √ | √ | √ |  | |
| 161388 | Virgaureagenin G | high | √ |  | √ | √ |  | |
| 21668841 | Onjisaponin A | high | √ | √ | √ | √ | √ | |
