# Supplementary material for: Network Pharmacology-Based Systematic Analysis of Molecular Mechanisms of Dingji Fumai Decoction for Ventricular Arrhythmia
Source: Evid Based Complement Alternat Med. 2021 May 8;2021:5535480. doi: 10.1155/2021/5535480 (PMC8128550; doi:10.1155/2021/5535480)
Supplement: Supplementary Materials — Table S1: comparison of Chinese medicine names and Latin names. Table S2: details of qualified compounds in various herbs. Figure S1: molecular docking modules. [file 5535480.f1.zip › 5535480.f1/Tbale S1.docx]

| Table S1. The comparison of Chinese medicine names and Latin names. | |
| --- | --- |
| Chinese name | Latin name |
| Chuanxiong | Chuanxiong Rhizoma |
| Dazao | Jujubae Fructus |
| Fuling | Poria Cocos (Schw.), Wolf |
| Guizhi | Cinnamomi Ramulus |
| Hehuanpi | Silktree Albizia Bark |
| Longgu | Osdraconis (Fossiliaossiamastodi) |
| Muli | Ostrea Gigas Thunberg |
| Suanzaoren | Ziziphi Spinosae Semen |
| Yuanzhi | Radix Polygalae |
| Gancao | Licorice |
